# Co-haplotyping symbiont and host to unravel invasion pathways of the exotic pest *Halyomorpha halys* in Italy

**DOI:** 10.1038/s41598-020-75519-2

**Published:** 2020-10-28

**Authors:** Isabel Martinez-Sañudo, M. Alejandra Perotti, Davide Scaccini, Alberto Pozzebon, Laura Marri, Luca Mazzon

**Affiliations:** 1 Department of Agronomy, Food, Natural resources, Animals and Environment (DAFNAE), University of Padua, Padua, Italy; 2 Ecology and Evolutionary Biology Section, School of Biological Sciences, University of Reading, Reading, UK; 3 Department of Life Sciences, University of Siena, Siena, Italy

**Keywords:** Population genetics, Entomology, Symbiosis, Invasive species

## Abstract

The brown marmorated stink bug *Halyomorpha halys* (Stål) is a globally invasive species that harbours the primary bacterial symbiont ‘*Candidatus* Pantoea carbekii’. In this work, *P. carbekii* was used as another genetic marker to investigate the biodiversity and biogeographical patterns of this important pest, in native and newly invaded areas, especially in Italy. The correlation between the genetic structure of the symbiont and that of its host was studied through the analyses of one bacterial and one host marker, the putative pseudogene ΔybgF and the mitochondrial gene *COI*, respectively. As a result, five new *P. carbekii* haplotypes were identified, and an association pattern between host-symbiont haplotypes was observed. Host species showed higher haplotype diversity than symbiont, which can be expected in a long term host-symbiont association. Populations from the north-eastern Italy showed the highest values of genetic diversity for both markers, highlighting that this particular Italian area could be the result of multiple ongoing introductions. Moreover, some of the symbiont-host haplotypes observed were shared only by populations from north-eastern Italy and native areas, especially Japan, suggesting further introductions from this native country to Italy. Overall, our findings improve the understanding of the potential origin of multiple accidental introductions of *H. halys* in Italy.

## Introduction

Symbiotic bacteria, especially primary or obligatory symbionts (p-symbionts) have the potential to be used as genetic markers to investigate host biodiversity and biogeographical patterns^1–3^. P-symbionts are transmitted vertically, from mother to offspring, and may accurately mirror the history of their insect hosts. This ultimately helps, in the case of invasive host species by shedding light on their invasion pathways^2,4^. Bacterial symbionts can be used as reliable markers, showing even higher resolution than their hosts^3,5,6^.

During the last century, biological invasions by exotic pests into new areas, generally facilitated by increased international trade and human movement, have caused costly economic damages in agriculture and forestry. Any new venue of information regarding the invasion pathways of a pest may help understanding its colonizing capacity, adaptability and behavior^7–9^, and consequently shape defining new control strategies.

The highly invasive brown marmorated stink bug *Halyomorpha halys* (Stål) (Hemiptera: Pentatomidae), harbours the primary, vertically transmitted symbiont ‘*Candidatus* Pantoea carbekii’ (hereafter referred to as *Pantoea carbekii*)^10^. Compared to free-living bacteria of the gammaprotobacteria, *P. carbekii* genome has experienced a consistent reduction, like other p-symbionts, but still provides its host with essential nutrients, vitamins, cofactors and protection of the most vulnerable stages of early development (1st nymphal stages)^11^. *Pantoea carbekii* is highly stress tolerant, especially once secreted to cover the eggs, by its unique biofilm-formation properties, securing host offspring survival^11^.

*Halyomorpha halys* is native to Southeast Asia but has recently invaded North America and Europe causing substantial damage to agricultural crops and creating nuisance to residents in rural and urban areas^12–14^. In Europe, since its first occurrence in Switzerland in 2004^15^, it has rapidly spread to almost other 17 European countries^16–33^, as well as to Russia, Abkhazia and Georgia^34,35^. Based on the newly reported distribution points and recent models of expansion, its dispersal will likely continue in the coming years^36,37^.

Several efforts have been made to identify the origin and movements of *H. halys* across the continents using genetic markers. Although most studies have focused on the use of the mitochondrial Cytochrome Oxidase I (*COI*) and Cytochrome Oxidase II (*COII*) genes^23,37–42^, one laboratory has attempted to resolve the genetic structure and origin considering some pseudogenes of the symbiont, *P. carbekii*, as genetic markers (*e.g*. ΔybgF)^3^. However, the bacterial marker did not explicitly incorporate geographic information among populations probably due to the limited sampling, especially in Europe^3^.

In this work, the genetic structure of the primary symbiont of populations of *H. halys* from native (China, Japan and South Korea) and newly invaded areas (United States, Italy and Hungary) was investigated utilizing the putative pseudogene ΔybgF. Special attention was given to invasive populations of north-east Italy, where high levels of genetic variability of the insect host have been recently reported^39,43^. The resulting data from the ΔybgF (bacterium marker) analysis was combined with the genetic structure of their insect hosts, for which the mitochondrial gene *COI* was used. Both native and newly introduced populations were studied to elucidate the pathways of expansion of the pest across the invaded area.

## Results

### Genetic diversity of the p-symbiont, *P. carbekii*

A total of 194 (2–18 individuals/population) high quality sequences of ΔybgF were obtained. For quality assessment of the sequences, the chromatograms were analysed, and low quality regions found at the beginning and end of each one were trimmed, resulting in high quality sequences 206 bp long. All sequences showed high identity (> 99% sequence similarity) with the available sequences of *P. carbekii* in GenBank NCBI^3,11^. After alignment of the sequences obtained from this study, a total of six variable sites, and three parsimony-informative sites were identified.

Calculation of population diversity indices was limited to those populations represented by more than five samples. The haplotype diversity (H) of the p-symbiont per population ranged from 0 for KGJ (South Korea), and IFVM and IFVU (Italy), to 0.778 in IVeMo (Italy). Similarly, nucleotide diversity (π) ranged from 0% in KGJ (South Korea), IFVM and IFVU (Italy) and 0.66% in IVeMo (Italy) (Table 1).

**Table 1:** Descriptive statistics of the endosymbiont *Pantoea carbekii* and the host *Halyomorpha halys* with the bacterial and the mitochondrial marker respectively.

Country	Code	Symbiont *Pantoea carbekii*					Host *Halyomorpha halys*				
		n seq	Haplotypes (ΔybgF)	n hap	H	π (%)	n seq	Haplotypes (COI)	n hap	H	π (%)
China	CCD	7	P2(2), P1(4), P6(1)	3	0.6667 ± 0.1598	0.42	10	H1(4), H3(3), H7(1), H65(2)	5	0.7778 ± 0.0907	0.36
	CBH	8	P2(2), P1(5), P7(1)	3	0.6071 ± 0.1640	0.38	10	H1(7), H3(2), H64(1)	3	0.5111 ± 0.1643	0.11
	CSD	5	P2(1), P1(4)	2	0.4000 ± 0.2373	0.19	7	H1(5), H3(1), H13(1)	3	0.5238 ± 0.2086	0.12
Japan	JIT	12	P2(3), P3(8), P5(1)	3	0.5303 ± 0.1359	0.31	13	H23(1), H53(1), H57(4), H60(2), H61(1), H62(4)	6	0.8333 ± 0.0714	0.38
South Korea	KGJ	10	P2(10)	1	0.0000 ± 0.0000	0.00	12	H2(1), H22(9), H63(1), H66(1)	4	0.4545 ± 0.1701	0.10
	KSG	3	P2(3)	1			6	H2(2), H22(4)	2	0.5333 ± 0.1721	0.10
Italy	IEB	4	P2(1), P1(3)	2			4	H1(3), H3(1)	2		
	IEP	16	P2(6), P1(10)	2	0.5000 ± 0.0741	0.24	17	H1(9), H3(4), H8(2), H52(1), H54(1)	5	0.6838 ± 0.0986	0.36
	ILoC	9	P2(6), P1(3)	2	0.5000 ± 0.1283	0.24	7	H1(3), H3(3), H8(1)	3	0.7143 ± 0.1267	0.23
	ILoL	8	P2(4), P1(4)	2	0.5714 ± 0.0945	0.28	10	H1(5), H3(5)	2	0.5556 ± 0.0745	0.11
	IFVM	9	P1(9)	1	0.0000 ± 0.0000	0.00	9	H1(9)	1	0.0000 ± 0.0000	0.00
	IFVU	6	P1(6)	1	0.0000 ± 0.0000	0.00	6	H1(6)	1	0.0000 ± 0.0000	0.00
	ILiT	3	P2(3)	1			4	H1(1), H3(1), H52(1), H54(1)	4		
	ITAR	15	P2(7), P1(6), P3(2)	3	0.6476 ± 0.0716	0.37	15	H1(8), H3(4), H23(1), H53(2)	4	0.6667 ± 0.0991	0.47
	IVeC	5	P1(3), P3(2)	2	0.6000 ± 0.1753	0.59	6	H1(4), H53(2)	2	0.5333 ± 0.1721	0.77
	IVeL	12	P2(5), P1(4), P3(3)	3	0.7121 ± 0.0691	0.43	11	H1(5), H3(1), H53(1), H23(1), H54(1), H58(1), H59(1)	7	0.8182 ± 0.1191	0.67
	IVeN	11	P2(5), P1(2), P3(4)	3	0.6909 ± 0.0861	0.41	11	H1(2), H3(1), H23(2), H53(5), H58(1)	5	0.7818 ± 0.1073	0.71
	IVeMo	10	P2(1), P1(4), P3(3), P4(2)	4	0.7778 ± 0.0907	0.66	11	H1(5), H53(4), H40(2)	3	0.6909 ± 0.0861	0.82
USA	UCCV	6	P2(4), P1(2)	2	0.5333 ± 0.1721	0.26	8	H1(2), H3(6)	2	0.4286 ± 0.1687	0.09
	UGT	2	P1(2)	1			2	H1(2)	1		
	UOS	15	P2(4), P1(11)	2	0.4190 ± 0.1132	0.20	16	H1(3), H3(1), H23(1), H56(1), H67(1), H79(1), H1017(8)	7	0.7417 ± 0.1053	0.58
Hungary	HCB	18	P2(7), P1(11)	2	0.5033 ± 0.0639	0.25	24	H1(21), H3(3)	2	0.2283 ± 0.1021	0.05
Tot		194					219				

In brackets the number of individuals bearing each haplotype. H: haplotype diversity, π (%): nucleotide diversity.

**Figure 1:**
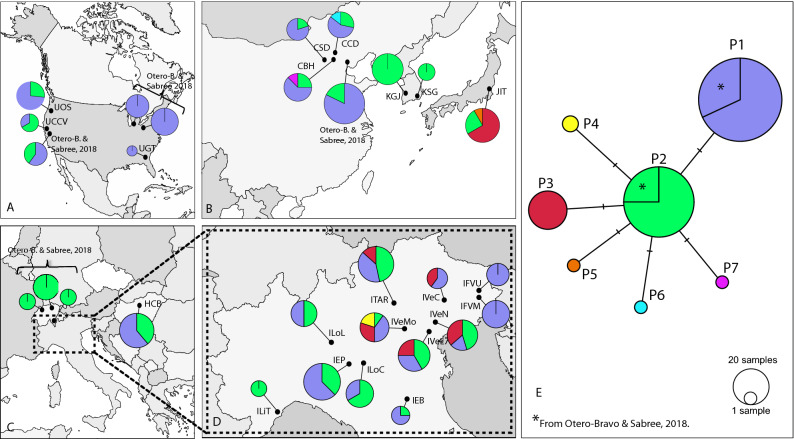
Proportional geographical distribution of *Pantoea carbekii* haplotypes using the pseudogene ΔybgF as a marker across sampled populations in invaded areas: USA (**A**), Europe (**C**) and north Italy (**D**) and native areas (**B**). TCS network of *P. carbekii* haplotypes constructed in PopART 1.7 is represented in (**E**). Each haplotype is represented by a circle, and the area of the circle is proportional to its frequency. The colours represent differences in geographic distribution, and hatch mark is a single mutation. Maps source: https://d-maps.com.

A statistical parsimony network which was built using our dataset and publicly available nucleotide sequences (NCBI), revealed seven different bacterium haplotypes. Five haplotypes of them (P3, P4, P5, P6, and P7) have been retrieved for the first time in this study (Fig. 1). Haplotype P2 was separated by only one mutational step from all the other haplotypes found (Fig. 1). Haplotypes P1 and P2, were the most common haplotypes showing the highest number of sequences: N_P1_ = 100 (38%) and N_P2_ = 138 (52%), respectively. They were followed in numbers by haplotype P3 (N = 22), haplotype P4 (N = 2), and the remaining haplotypes, P5, P6, and P7, with one sequence each. Haplotype P1 was the most frequent among populations from the native area (China) and from the newly invaded areas (Fig. 1). Haplotype P2 was found in all the studied populations, except for three Italian populations, IVeC, IFVM and IFVU, and the USA populations UGT. Haplotype P3 was present in five populations from north-eastern Italy (ITAR, IVeC, IVeL, IVeN, and IVeMo), and in the population from Japan (JIT). Haplotype P4 was recorded in two samples from the Italian population IVeMo. Lastly, haplotypes P4, P5, P6 and P7 were rare and limited to samples collected from the native area, except P4 that was recorded in two samples from the Italian population IVeMo (Fig. 1).

### Genetic diversity of the host, *H. halys*

After quality checking and trimming the sequences, a fragment of 490 bp of *COI* gene was obtained for all 219 specimens (2–24 individuals/population) representing the 22 populations of *H. halys* object of this study. The rarefaction curve reached an asymptote at 0% difference among sequences (Supplementary Figure S2). All sequences showed a similarity > 99% with the *H. halys COI* gene available in the GenBank database. Twenty polymorphic sites were identified by the alignment of the obtained sequences and resulted parsimony informative. As in the case of *P. carbekii* data, the diversity indices of the host were calculated only for those populations for which more than five samples were available. Diversity indices ranged between 0.00 and 0.83 for the haplotype diversity H, and between 0% and 0.82% for the nucleotide diversity π (Table 1). The population from Japan (JIT), followed by the IVeL population from Italy, showed the highest H values, while the population IVeMo from Italy displayed the highest π value (Table 1).

A haplotype network, including a total of 1153 sequences, was built by combining *H. halys* sequences from GenBank (Supplementary Table S1) and from our dataset. Hundred and twenty-three haplotypes were displayed in the network: 97 included only sequence records in GenBank, 21 shared sequences from both GenBank and this study and five were exclusively from this study (Supplementary Figure S1).

The network presented two frequent and spread haplotypes (H1 and H3) plus many rare haplotypes. Haplotypes H1 and H3 included samples from all countries of the invaded areas (Canada, USA, Croatia, France, Greece, Hungary, Italy, Romania and Switzerland), and few samples from the native areas in China (Supplementary Figure S1). A geographic structure of the native countries can be observed in the haplotypes network. Haplotypes H2 and H22 (samples from South Korea), and haplotypes H1 and H3 (samples from China) were separated by only one mutational step. Several rare haplotypes were connected in a star-shape way to the former haplotypes (Supplementary Figure S1). Japanese samples were associated with many rare haplotypes scattered only in a portion of the network. Some of them were shared with populations from north-eastern Italy, such as IVeN, IVeMo, IVeC (Supplementary Figure S1).

Tajima’s D and Fu’s values found in *H. halys* populations from native countries (China, Japan and South Korea) and two newly invaded populations (Switzerland and France) rejected the null hypothesis of neutrality, suggesting a past population expansion of *H. halys* in these areas (Supplementary Table S2). The mismatch distribution of the native populations from China and South Korea and the introduced populations from France, Hungary and Switzerland showed unimodal curves (Supplementary Figure S3) but only populations from China showed SSD and raggedness index (r) values that did not reject a sudden expansion model (Supplementary Figure S3). The remaining populations showed multimodal curves, SSD and r-values that rejected the sudden expansion model.

### Patterns of association of host–symbiont haplotypes

**Figure 2:**
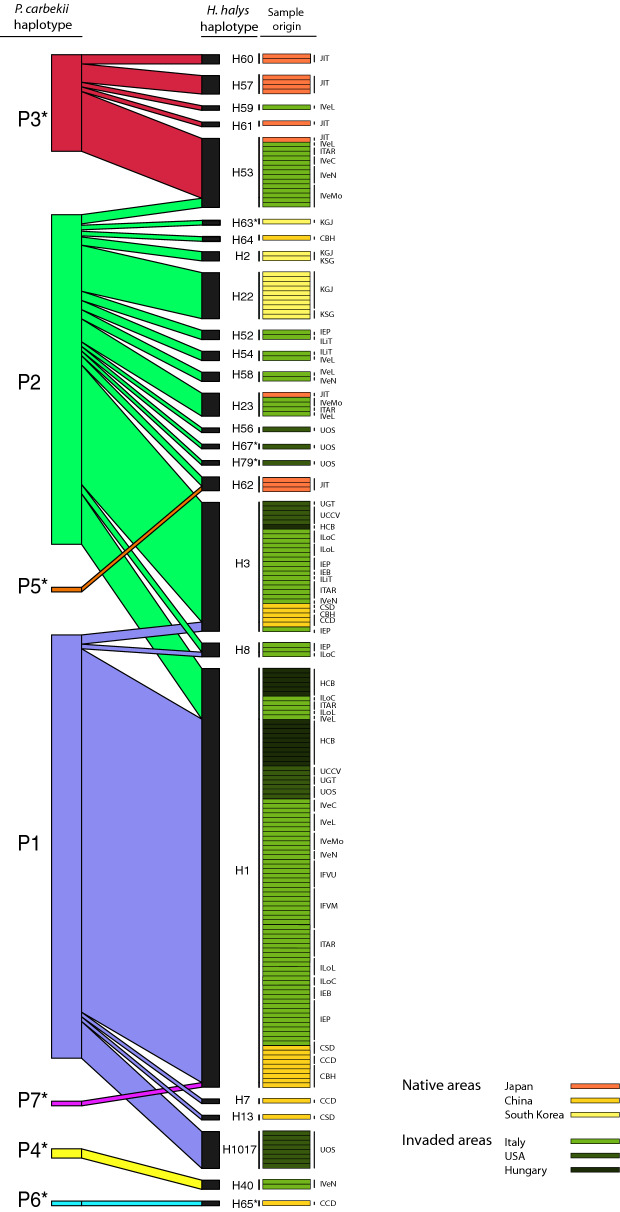
Weighted bipartite networks of interactions between the symbiont *Pantoea carbekii* (left bars) and the host *Halyomorpha halys* (black bars). The width of the bars reflects the relative frequency of *P. carbekii* and *H. halys* haplotypes, respectively. The width of the lines connecting bars indicates the frequency with which a symbiont haplotype was found in a given host haplotype. The geographical origin of each sample is represented by horizontal rectangles. Codes reported on the right refer to the population origin (see Table 2). Asterisk * indicates new haplotypes found in this study.

A bipartite interaction matrix between the haplotypes of the host and the symbiont was built considering only the samples in which the sequence of both the symbiont and the host were obtained (Fig. 2). The network of interactions consisted of seven *P. carbekii* haplotypes with 25 *H. halys* haplotypes. The specialization index H2′ showed significantly high network specialization (H2′ = 0.71, Z-score = 24.36). The NODF value, significantly lower than expected, did not exhibit a nested pattern (NODF = 23.61, Z-score = − 8.10) confirming the specificity between symbiont and host haplotypes.

The most frequent *P. carbekii* haplotype, P1, was more often associated with H1 host haplotype. This association was observed both in samples collected in China and in all the new invaded regions considered in this work, which were twelve populations from Italy, three from USA and one from Hungary (Fig. 2).

*Pantoea carbekii* haplotype P2 was correlated with the highest number of host haplotypes (n = 16) coming from native (China, Japan and Korea) and newly invaded areas (Italy, Hungary and USA); notably, in the majority of these associations (11 out of 16), the host haplotypes were exclusively associated with the symbiont haplotype. Host haplotype H3 was most frequently associated with haplotype P2, and it was present in samples from China and several populations from the newly invaded areas: six populations from Italy, two populations from USA, and one from Hungary (Fig. 2).

*Pantoea carbekii* haplotype P3 was shared with five host haplotypes including samples from Japan and Italy, more specifically the four north-eastern Italy populations of ITAR, IVeC, IVeL, and IVeN. While *P. carbekii* haplotypes P4 and P6 were strictly associated to host haplotype H40 and H65, respectively. The symbiont-host association P4-H40 was present in samples from China and north-east Italy, while P6-H65 was only found in one sample from China (Fig. 2). Finally, P5 was associated with the host haplotype H62 represented by samples from Japan and symbiont haplotype P7 was associated with the host haplotype H1.

The majority of host haplotypes (20 out of 25) were exclusively associated to one symbiont haplotype, and only five host haplotypes showed associations with more than one. Host haplotype H53 was mainly associated with symbiont haplotype P3, except for 2 samples out of 15, which resulted to be associated with haplotype P2. Similarly, host haplotype H3 was mostly associated with haplotype P2, and only 2 samples were associated with haplotype P1. In general, H1 harboured p-symbiont haplotype P1, fewer samples (N = 11) carried P2, and only one H1 sample was associated with haplotype P7. Host haplotypes H62 and H8 were associated with P2 and P5, and with P2 and P1, respectively (Fig. 2).

## Discussion

This study, based on populations of *H. halys* from six countries, native and newly invaded areas, has unravelled five new haplotypes of its p-symbiont *P. carbekii*, increasing the total number of known haplotypes from two^3^ to seven. Perhaps the most intriguing outcome of this study, and of a similar one conducted by Otero-Bravo & Sabree^3^ (they used *COII* and 12S genes) was the finding of a higher haplotype diversity in the host species, *H. halys* than in the symbiont.

It is assumed that increased genetic variability of invasive species in the invaded areas is associated with multiple introductions^44^. Likely, populations of north-east Italy could be the result of multiple, still ongoing, introductions from either different parts of the native areas and even other invaded regions.

The whole dataset allowed to observe some dispersion patterns of the invasive pest *H. halys*. The north-eastern Italian populations showed symbiont-host haplotypes associations that differed from the rest of north Italian populations, which support the presence of multiple *H. halys* invasions from different origins. Haplotypes P1 and P2, already detected by Otero-Bravo & Sabree^3^, included the highest number of sequences: 100 (38%) and 138 (52%) of the dataset including both sequences of this study and NCBI, respectively. The topology of the host network matched with the one obtained by Cesari et al*.*^39^ and Valentin et al.^41^ that was characterized by the presence of two frequent and spread haplotypes (H1 and H3) and many rare haplotypes.

The p-symbiont haplotype P1, strongly associated with H1, was the most spread haplotype across the invaded areas including Europe and USA and it was found in all the Chinese populations object of this study. The symbiont-host association P1-H1 seems the most successful combination for invasiveness, or the most widespread combination. Its presence in the newly invaded areas may be attributed to a single or few introductions of H1 carrying P1, probably from China, and from there it could have spread across the globe. However, we cannot exclude that this widespread association may be the result of multiple introductions in different parts of the world by many samples sharing the same Chinese combination of haplotypes (P1-H1), possibly due to the commercial trades. Overall, it could be due to a single small initial invasive population with haplotypes P1-H1 or multiple invasions from the same source. After a shipment from USA to Germany^20,45^, the documented interception of *H. halys* in Europe, being all haplotype H1, suggests that European populations with haplotypes P1-H1, could have arrived from USA instead of coming directly from China^46^. The presence of the host haplotype H1 in Italy has been considered the result of an introduction event (from China or USA) different from the one reported for the first time in 2004 in Europe, Switzerland^39,46^. Indeed, the first populations found in Switzerland hosted the p-symbiont haplotype P2^3^, supporting that the P1-H1 association found in samples collected in Italy might have resulted from a different introduction event either from China or USA.

P-symbiont haplotype P2, was found in association with the highest number of host haplotypes, and more frequently linked to the host haplotype H3. The association P2-H3 was detected in samples collected within the native areas in China, and in the early invaded areas of Switzerland, Italy (mostly north-western areas), USA, and Hungary. The presence of P2-H3 in Italy could have resulted from its spread from China to Switzerland and, subsequently, from there to the North of Italy, by active and passive movement^39, 41, 46^. Active dispersal involves movement of the entire organism through its own ability (*e.g.* walking, flying, falling) while in passive dispersal, an organism uses different mechanisms (*e.g.* wind and human-mediated transport) to exploit new habitats^47,48^. Other symbiont-host associations, like P2-H52, P2-H54, including samples from both China (Shandong province) and north-west Italy, support the hypothesis that they came from China, in particular north-eastern areas of the native country. Moreover, host samples with haplotype H23 harbouring the p-symbiont haplotype P2 found in a number of populations from north-east Italy, ITAR, IVeL and IVeN, and Japan, highlight their possible recent arrival in Italy from Japan. In Oregon, the symbiont haplotype P2 was associated with two novel host haplotypes, H67 and H79, as well as with host haplotypes H56 and H23, which included also samples from Japan and Italy. The results pointed out the possibility that new introductions of *H. halys* from native or even newly invaded areas can take place continuously. In recent studies it was observed that after years of low genetic variability in USA populations^23,38,42^, new *H. halys* haplotypes have been found, suggesting that new invasion events have recently occurred in USA^37,39,42^.

P-symbiont haplotype P3 was the most frequent among the five new symbiont haplotypes reported for the first time in this study. It was mostly associated with host haplotypes H53 occurring in populations of north-eastern Italy and Japan; adding to further introductions of *H. halys* from Japan to Italy. While haplotype P4 was strictly associated to host haplotype H40 from China and north-eastern Italy, strengthening the possibility of multiple introductions from China to Italy.

These findings, combined with the information provided by other authors^20,39–42^ shed new light on the dispersion patterns of *H. halys* into the new invaded regions of north-east Italy. It is likely that (1) one or more introduction events of P1-H1 from China (and/or USA) to north Italy have taken place, (2) that one or more introduction events of P2-H3 came first from China to Switzerland, and, subsequently, to north-west Italy and, (3) that one or more introduction events of P3-H53 and P2-H23 came directly from Japan to north-east Italy.

The complex network route of introduction and the high diversity of *H. halys* retrieved in samples collected in north-eastern Italy, could be explained by a large national and international commercial trade in this area. This can be supported by the presence of two of the largest and busiest Italian ports importing high volume of solid commodities^49^. The ability of *H. halys* to travel, joining commercial or domestic transport and with any type of goods, being often undetected during phytosanitary checks facilitates its accidental introductions into new territories^50–52^. Moreover, a study conducted by Zhu et al.^40^ proposes that *H. halys* populations from northern China possess high dispersal ability and/or adaptability. The former characteristics, together with the available suitable climate space in the invaded areas, suggest that the pest may expand northward to higher latitudes^40^.

The high genetic diversity of the host, *H. halys*, which showed the existence of 25 haplotypes, interacting with only seven *P. carbekii* (p-symbiont) haplotypes can be expected from a long term host-symbiont association.

The stink bug family (Pentatomidae) carry several lineages of gut symbionts standing at different evolutionary stages^53^. Studies on molecular evolution rates of these pentatomid symbionts have revealed the existence of reduced genomes (due considerable gene losses), coupled with high AT composition and accelerated evolution, a characteristic of stable host-symbiont associations, and not observed in frequently promiscuous symbionts (for example, exhibiting frequent horizontal transmission due to exposure to free living bacteria)^53^. A phylogeny carried out on pentatomid symbionts, showed that the stable lineages are conserved and host species specific^53^. This same study found that *P. carbekii* has an accelerated molecular evolution, being one of the few uncultivable symbionts within the stink bugs clade. This pattern is generally observed and expected from endosymbionts, or intracellular bacterial symbionts which have reached a stable, old host association living in the protected intracellular environment^53,54^. P-symbionts have well established ancient associations that are also explained by the provision of bacteriomic or mycetomic organs by their host^55^. *Halyomorpha halys*, as all other pentatomid bugs, provides a unique bacteriomic structure, consisting of midgut caecal crypts in the distal end of the gut^10^. The physiological processes involving this organ include the selective uptake of the unique bacterial symbiont species, being the organ able to discriminate from non-symbiotic bacterial species^56^. This is a feature of long-standing, ancient host-symbiont associations.

According to the Muller’s ratchet hypothesis, p-symbionts (in bacteria from small populations with low recombination rates) increasingly acquire deleterious mutations, leading to sharp extinction; but this is not happening^54^. Instead, p-symbionts seem to be exposed to selective pressure over time, removing deleterious mutations with reduction in base substitutions; this is dominating in older or ancient associations, slowing down the molecular evolutionary rates of the symbionts^54^. As *H. halys* is a ~ 160 million years old bug^57^, the association with *Pantoea* might have been established since the emergence of the clade, evolving for as long as the polyphagous host exists. Results from this work and previous research^3^ suggest that *P. carbekii* is under strong selective pressure, consequently keeping a low diversity (haplotypes). *Pantoea carbekii* stability makes possible the generalist polyphagous habits of *H. halys*. This host can rely on a stable p-symbiont that secures colonization, by providing nutrients, vitamins, and crucially securing survival of vulnerable developmental stages, regardless of the type of plant species and/or new environments invaded.

*Halyomorpha halys* is highly flexible, rapidly adapting to new and varied environments. The pest has high genetic diversity, observed in the multitude of haplotypes revealed. This genetic plasticity is not new, it has been observed in populations from the native and original areas, likely central China^40^, and seems to be shaped since the milder Pleistocene climate of East Asia. Latest research modelling the Pleistocene history of *H. halys*, estimated that the rapid expansions that took place in the past have occurred towards the Last Glacial Maximum. The same study has predicted future expansions toward higher latitudes, as it is already happening, in US and Europe^40^.

This study improves the basic knowledge of the symbiotic relationship and provides new insights about potential origin of accidental introductions of the exotic species in Italy. Considering the impact that free trade exhibits on the rapid expansion of alien pests, a specific strategic plan might be developed in order to reduce the invasion risk by this pest. The results herein provide a framework for future researches that could help to optimize specific monitoring programs of material trade between national and international authorities.

## Material and methods

### Sample collection and preparation

Adults of *H. halys* were collected during three years (2017–2019) in six countries; three representing their native ranges (China, South Korea and Japan) and three new invaded areas (United States, Italy and Hungary). Extensive sampling was conducted in Italy encompassing the northern regions where the pest is mostly present (Emilia-Romagna, Friuli-Venezia Giulia, Liguria, Lombardy, Trentino-Alto Adige and Veneto). In total, 22 localities (populations) were sampled (Table 2).

**Table 2:** Collection sites and code of *Halyomorpha halys* populations analysed.

Country	Code	Region	Locality	Latitude	Longitude
China	CCD	Jingjinji, Beijing	Changping District	40°18′16.3ʺ N	116°11′23.5ʺ E
	CBH		Beijing, Huairou	40°24′39.5ʺ N	116°17′55.3ʺ E
	CSD		Shijingshan District	39°56′29.0ʺ N	116°10′19.4ʺ E
Japan	JIT	Ibaraki	Tsuchiura-shi	36°10′17.6ʺ N	140°09′46.4ʺ E
South Korea	KGJ	Gimje	Jeollabuk	35°52′12.7ʺ N	126°57′48.5ʺ E
	KSG	Sacheon	Gyeongsangnam	35°03′29.3ʺ N	128°05′36.7ʺ E
Italy	IEB	Emilia Romagna	Bologna, Vill. di Castenaso	44°30′34.0ʺ N	11°26′13.1ʺ E
	IEP		Piacenza	45°02′14.7ʺ N	9°43′49.2ʺ E
	IFVM	Friuli-Venezia Giulia	Moruzzo	46°07′20.4ʺ N	13°07′04.5ʺ E
	IFVU		Udine	46°01′13.2ʺ N	13°01′45.9ʺ E
	ILiT	Liguria	Toirano	44°07′36.8ʺ N	8°11′58.6ʺ E
	ILoC	Lombardy	Cremona	45°08′37.5ʺ N	9°59′21.4ʺ E
	ILoL		Lodi	45°24′02.2ʺ N	9°26′19.4ʺ E
	ITAR	Trentino-Alto Adige	Rovereto	45°53′31.3ʺ N	11°02′49.9ʺ E
	IVeC	Veneto	Conegliano	45°53′05.2ʺ N	12°16′36.7ʺ E
	IVeL		Legnaro	45°20′49.0ʺ N	11°57′26.2ʺ E
	IVeMo		Montecchio Maggiore	45°33′05.5ʺ N	11°23′59.7ʺ E
	IVeN		Noale	45°29′43.5ʺ N	12°03′26.8ʺ E
USA	UCCV	California	Central Valley	37°44′49.2ʺ N	121°54′57.2ʺ W
	UGT	Georgia	Tifton	31°28′26.4ʺ N	83°31′50.1ʺ W
	UOS	Oregon	Salem	44°53′59.7ʺ N	123°06′37.6ʺ W
Hungary	HCB	Central Hungary	Budapest	47°28′49.3ʺ N	19°02′27.1ʺ E

Collected samples were kept in 96% ethanol and shipped to the laboratory. The insects were morphologically identified following the characters described in Maistrello et al.^58^ and subsequently stored in 96% ethanol at − 20 °C until being processed.

Before performing the molecular studies, the abdomen of the insects was dissected under a stereomicroscope in a laminar flow hood using sterile equipment and sterile water. The intestinal tract (V4 region) harbouring the symbionts was extracted, transferred to Eppendorf tubes and kept in 96% ethanol at − 20 °C for further analysis.

### Genetic analysis

Extraction of DNA from each individual sample was performed using the Qiagen DNeasy Blood & Tissue Kit (Qiagen, Valencia, CA, USA) following the manufacturer’s instructions. The putative pseudogene ΔybgF previously used by Otero-Bravo & Sabree^3^ was selected to analyse the genetic variability of the p-symbiont. Same samples were used both for amplifying *P. carbekii* pseudogene ΔybgF, and *H. halys* mitochondrial cytochrome oxidase I (*COI*) gene.

Primers dYbg-F and dYbg-R^3^ were used in order to amplify the putative pseudogene ΔybgF in 20 μl reactions (1 × PCR Go Taq Flexi buffer—Promega, 2.5 mM MgCl_2_, 0.1 mM dNTPs, 0.5 μM of each primer, 0.5 U of Taq polymerase—Promega, 2 μl DNA template). Thermal cycling conditions were 3 min at 95 °C followed by 30 cycles of 95 °C for 30 s, 50 °C for 30 s, and 72 °C for 30 s with a final extension of 72 °C for 2 min.

Genetic diversity of the host *H. halys* was studied using a region of the mitochondrial DNA corresponding to a fragment of the Cytochrome C Oxidase subunit I (*COI*) which was amplified using the universal primer pairs LCO-1490/HCO-2198, following procedures as in Folmer et al.^59^.

PCR products were checked via electrophoresis on 1.0% agarose gels stained with SYBR Safe nucleic acid stain (Invitrogen), purified using Exonuclease and Antarctic Phosphatase (GE Healthcare) and sequenced at the BMR Genomics Service (Padua, Italy). For the sequences obtained, the chromatograms were quality checked in Geneious prime 2020.1.2 (https://www.geneious.com)^60^ and trimmed before further analysis. Low quality sequences were not included in the analysis.

### Data analysis

Sequences were edited and aligned using MEGA X^61^. A GenBank BLAST analysis of each of the sequences obtained was run through the NCBI website (www.ncbi.nlm.nih.gov) to assess the identity of the sequences. In order to estimate the adequacy of sampling a rarefaction curve of the *COI* data set was generated using DOTUR 1.53^62^ considering the number of OTUs obtained at the 0% level of sequence divergence. If rarefaction curves reach an asymptote it is considered that most of the actual diversity have been sampled. Haplotype and nucleotide diversity of symbiont and host markers (each population), were calculated with Arlequin 3.5^63^ using a Kimura 2-parameters model. The symbiont haplotype names in this study follow the nomenclature given by Otero-Bravo & Sabree^3^.

Sequences of the same markers available in the NCBI database were retrieved and added to this study datasets, in order to build haplotype networks for both the symbiont and the host. Statistical parsimony haplotype networks were inferred using the software PopART 1.7^64^ and TCS 1.21^65^.

The demographic history of *H. halys* was inferred within each country, using the *COI* dataset, through the Tajima’s D and Fu’s Fs test and mismatch distributions of the pairwise genetic differences using Arlequin 3.5. The raggedness index (r) was used to quantify the smoothness of mismatch distribution and the sudden expansion model was tested through the analysis of the sum of square deviations (SSD) representing the modality of the distribution, obtaining the corresponding P values with a parametric bootstrap approach (10,000 replicates).

In addition, associations among symbiont-host haplotypes were represented in a bipartite network. The specialization index (H2′) was used to determine the overall network specialization. Networks composed of specialized associations (*e.g.* symbiont-host association) show high H2′ values, whereas networks composed of not specialized associations show H2′ value of zero^66,67^. Moreover, the Nestedness Overlap and Decreasing Fill (NODF) index was used to detect and quantify the level of nestedness. Increasing values of NODF show increasing nestedness in the network^68^. Graph and metrics were generated by the bipartite package^69^ in R 3.0.1 software (R Development Core Team—https://www.r-project.org).

## Supplementary information

Supplementary Information

## Data Availability

All data used in this paper is available through GenBank accession numbers MT773135—MT773141 for the symbiont sequences and MT765125—MT765150 for the host sequences. Accession numbers of downloaded sequences used in the analysis are provided within a supplementary table sorted by haplotype (Supplementary Table S1).
