# Supplementary material for: Co-haplotyping symbiont and host to unravel invasion pathways of the exotic pest Halyomorpha halys in Italy
Source: Sci Rep. 2020 Oct 28;10:18441. doi: 10.1038/s41598-020-75519-2 (PMC7595193; doi:10.1038/s41598-020-75519-2)

Co-haplotyping symbiont and host to unravel invasion pathways of the exotic pest *Halyomorpha halys* in Italy

Isabel Martinez-Sañudo[1*], M. Alejandra Perotti[2], Davide Scaccini[1], Alberto Pozzebon[1], Laura Marri[3], Luca Mazzon[1]

Author affiliations:

[1]Department of Agronomy, Food, Natural Resources, Animals and Environment, University of Padova, Italy;

[2]Ecology and Evolutionary Biology Section, School of Biological Sciences, University of Reading, Reading, UK;

[31]Department of Life Sciences, University of Siena, Siena, Italy

[*]Corresponding author: isabel.martinez@unipd.it

Table S1. List of COI sequences of *Halyomorpha halys* retrieved from GenBank.

ID	Haplotype	Country	Authors
KX017396	H1	USA	Lee et al., 2018[42]
KX017395	H1	USA	Lee et al., 2018[42]
KX017394	H51	USA	Lee et al., 2018[42]
KX017393	H1	USA	Lee et al., 2018[42]
KX017392	H1	USA	Lee et al., 2018[42]
KX017391	H1	USA	Lee et al., 2018[42]
KX017390	H1	USA	Lee et al., 2018[42]
KX017389	H1	USA	Lee et al., 2018[42]
KX017388	H50	USA	Lee et al., 2018[42]
KX017387	H33	South Korea	Lee et al., 2018[42]
KX017386	H33	South Korea	Lee et al., 2018[42]
KX017385	H33	South Korea	Lee et al., 2018[42]
KX017384	H2	South Korea	Lee et al., 2018[42]
KX017383	H47	South Korea	Lee et al., 2018[42]
KX017382	H22	South Korea	Lee et al., 2018[42]
KX017381	H22	South Korea	Lee et al., 2018[42]
KX017380	H22	South Korea	Lee et al., 2018[42]
KX017379	H22	South Korea	Lee et al., 2018[42]
KX017378	H22	South Korea	Lee et al., 2018[42]
KX017377	H22	South Korea	Lee et al., 2018[42]
KX017376	H35	South Korea	Lee et al., 2018[42]
KX017375	H2	South Korea	Lee et al., 2018[42]
KX017374	H22	South Korea	Lee et al., 2018[42]
KX017373	H2	South Korea	Lee et al., 2018[42]
KX017372	H2	South Korea	Lee et al., 2018[42]

KX017371	H22	South Korea	Lee et al., 2018[42]
KX017370	H48	South Korea	Lee et al., 2018[42]
KX017369	H22	South Korea	Lee et al., 2018[42]
KX017368	H2	South Korea	Lee et al., 2018[42]
KX017367	H2	South Korea	Lee et al., 2018[42]
KX017366	H2	South Korea	Lee et al., 2018[42]
KX017365	H22	South Korea	Lee et al., 2018[42]
KX017364	H22	South Korea	Lee et al., 2018[42]
KX017363	H22	South Korea	Lee et al., 2018[42]
KX017362	H22	South Korea	Lee et al., 2018[42]
KX017361	H22	South Korea	Lee et al., 2018[42]
KX017360	H22	South Korea	Lee et al., 2018[42]
KX017359	H22	South Korea	Lee et al., 2018[42]
KX017358	H33	South Korea	Lee et al., 2018[42]
KX017357	H33	South Korea	Lee et al., 2018[42]
KX017356	H34	South Korea	Lee et al., 2018[42]
KX017355	H37	South Korea	Lee et al., 2018[42]
KX017354	H42	South Korea	Lee et al., 2018[42]
KX017353	H22	South Korea	Lee et al., 2018[42]
KX017352	H22	South Korea	Lee et al., 2018[42]
KX017351	H22	South Korea	Lee et al., 2018[42]
KX017350	H2	South Korea	Lee et al., 2018[42]
KX017349	H22	South Korea	Lee et al., 2018[42]
KX017348	H44	South Korea	Lee et al., 2018[42]
KX017347	H22	South Korea	Lee et al., 2018[42]
KX017346	H41	South Korea	Lee et al., 2018[42]
KX017345	H22	South Korea	Lee et al., 2018[42]
KX017344	H22	South Korea	Lee et al., 2018[42]
KX017343	H2	South Korea	Lee et al., 2018[42]
KX017342	H22	South Korea	Lee et al., 2018[42]
KX017341	H22	South Korea	Lee et al., 2018[42]
KX017340	H22	South Korea	Lee et al., 2018[42]
KX017339	H22	South Korea	Lee et al., 2018[42]
KX017338	H2	South Korea	Lee et al., 2018[42]
KX017337	H38	South Korea	Lee et al., 2018[42]
KX017336	H22	South Korea	Lee et al., 2018[42]
KX017335	H2	South Korea	Lee et al., 2018[42]
KX017334	H49	South Korea	Lee et al., 2018[42]
KX017333	H24	South Korea	Lee et al., 2018[42]
KX017332	H43	South Korea	Lee et al., 2018[42]
KX017331	H34	South Korea	Lee et al., 2018[42]
KX017330	H22	South Korea	Lee et al., 2018[42]
KX017329	H22	South Korea	Lee et al., 2018[42]
KX017328	H22	South Korea	Lee et al., 2018[42]
KX017327	H22	South Korea	Lee et al., 2018[42]
KX017326	H22	South Korea	Lee et al., 2018[42]
KX017325	H22	South Korea	Lee et al., 2018[42]

KX017324	H22	South Korea	Lee et al., 2018[42]
KX017323	H22	South Korea	Lee et al., 2018[42]
KX017322	H22	South Korea	Lee et al., 2018[42]
KX017321	H22	South Korea	Lee et al., 2018[42]
KX017320	H22	South Korea	Lee et al., 2018[42]
KX017319	H2	South Korea	Lee et al., 2018[42]
KX017318	H2	South Korea	Lee et al., 2018[42]
KX017317	H2	South Korea	Lee et al., 2018[42]
KX017316	H22	South Korea	Lee et al., 2018[42]
KX017315	H2	South Korea	Lee et al., 2018[42]
KX017314	H22	South Korea	Lee et al., 2018[42]
KX017313	H2	South Korea	Lee et al., 2018[42]
KX017312	H22	South Korea	Lee et al., 2018[42]
KX017311	H22	South Korea	Lee et al., 2018[42]
KX017310	H2	South Korea	Lee et al., 2018[42]
KX017309	H22	South Korea	Lee et al., 2018[42]
KP151524	H22	South Korea	Park & Lee, unpublished
JX548483	H25	USA	Tillman et al., 2015[70]
KM893988	H1	USA	Radulovici & Tiberio, unpublished
JX548487	H25	USA	Tillman et al., 2015[70]
JX548484	H25	USA	Tillman et al., 2015[70]
JX548485	H25	USA	Tillman et al., 2015[70]
JX548486	H25	USA	Tillman et al., 2015[70]
JX548488	H25	USA	Tillman et al., 2015[70]
JX548489	H25	USA	Tillman et al., 2015[70]
KC510116	H24	South Korea	Cho et al., unpublished
KF112038	H1	China, USA	Xu et al., 2014[38]
KF112039	H26	China, USA	Xu et al., 2014[38]
KF112040	H1	China	Xu et al., 2014[38]
KF273380	H1	France, Greece, Hungary, Switzerland	Gariepy et al., 2014[20]
KF273381	H2	China	Gariepy et al., 2014[20]
KF273382	H3	France, Greece, Hungary, Switzerland	Gariepy et al., 2014[20]
KF273383	H4	China	Gariepy et al., 2014[20]
KF273384	H5	China	Gariepy et al., 2014[20]
KF273385	H6	China	Gariepy et al., 2014[20]
KF273386	H7	China	Gariepy et al., 2014[20]
KF273387	H8	Switzerland, France	Gariepy et al., 2014[20]
KF273388	H9	Switzerland	Gariepy et al., 2014[20]
KF273389	H1	China	Gariepy et al., 2014[20]
KF273390	H11	China	Gariepy et al., 2014[20]
KF273391	H12	China	Gariepy et al., 2014[20]
KF273392	H13	China	Gariepy et al., 2014[20]
KF273393	H14	China	Gariepy et al., 2014[20]
KF273394	H15	China	Gariepy et al., 2014[20]
KF273395	H13	China	Gariepy et al., 2014[20]
KF273396	H17	China	Gariepy et al., 2014[20]
KF273397	H18	China	Gariepy et al., 2014[20]

KF273398	H19	China	Gariepy et al., 2014[20]
KF273399	H20	China	Gariepy et al., 2014[20]
KF273400	H21	China	Gariepy et al., 2014[20]
KF273401	H22	South Korea, Greece	Gariepy et al., 2014[20]
KF273402	H23	Japan	Gariepy et al., 2014[20]
KM401489	H3	Switzerland	Cesari et al., 2015[23]
KM401490	H3	Switzerland	Cesari et al., 2015[23]
KM401491	H8	Italy	Cesari et al., 2015[23]
KM401492	H1	Italy	Cesari et al., 2015[23]
KM401493	H3	Italy	Cesari et al., 2015[23]
KM401494	H3	Italy	Cesari et al., 2015[23]
KM401495	H3	Italy	Cesari et al., 2015[23]
KM401496	H3	Italy	Cesari et al., 2015[23]
KM401497	H3	Italy	Cesari et al., 2015[23]
KM401498	H3	Italy	Cesari et al., 2015[23]
KM401499	H3	Italy	Cesari et al., 2015[23]
KM401500	H1	Italy	Cesari et al., 2015[23]
KM401501	H1	Italy	Cesari et al., 2015[23]
KM401502	H1	Italy	Cesari et al., 2015[23]
KM401503	H1	Italy	Cesari et al., 2015[23]
KY930701	H1	Croatia	Sapina et al., 2018[71]
KM401504	H1	Italy	Cesari et al., 2015[23]
KM401505	H1	Italy	Cesari et al., 2015[23]
KY930700	H1	Croatia	Sapina et al., 2018[71]
KY930699	H1	Croatia	Sapina et al., 2018[71]
KM401506	H1	Italy	Cesari et al., 2015[23]
KM401507	H1	Italy	Cesari et al., 2015[23]
KM401508	H1	Italy	Cesari et al., 2015[23]
KM401509	H1	Italy	Cesari et al., 2015[23]
KM401510	H1	Italy	Cesari et al., 2015[23]
KM401511	H1	Italy	Cesari et al., 2015[23]
KM401512	H1	Italy	Cesari et al., 2015[23]
KM401513	H1	Italy	Cesari et al., 2015[23]
KM401514	H1	Italy	Cesari et al., 2015[23]
KM401515	H1	Italy	Cesari et al., 2015[23]
KM401516	H1	Italy	Cesari et al., 2015[23]
KM401517	H1	Italy	Cesari et al., 2015[23]
KM401518	H1	Italy	Cesari et al., 2015[23]
KM401519	H1	Italy	Cesari et al., 2015[23]
KM401520	H1	Italy	Cesari et al., 2015[23]
KM401521	H1	Italy	Cesari et al., 2015[23]
KM401522	H1	Italy	Cesari et al., 2015[23]
KM401523	H1	Italy	Cesari et al., 2015[23]
KM401524	H1	Italy	Cesari et al., 2015[23]
KM401525	H1	Italy	Cesari et al., 2015[23]
KM401526	H1	Italy	Cesari et al., 2015[23]
KM401527	H1	Italy	Cesari et al., 2015[23]

KM401528	H1	Italy	Cesari et al., 2015[23]
KM401529	H1	Italy	Cesari et al., 2015[23]
KM401530	H1	Italy	Cesari et al., 2015[23]
KR070748	H31	Greece	Gariepy et al., 2015[23]
KR070749	H30	Greece	Gariepy et al., 2015[23]
KR0707501	H32	Greece	Gariepy et al., 2015[23]
KR0707511	H33	Greece	Gariepy et al., 2015[23]
KU601517	H1	USA	Dhami et al., 2016[72]
KU601518	H1	USA	Dhami et al., 2016[72]
KU601519	H1017	USA	Dhami et al., 2016[72]
KU601521	H68	Japan	Dhami et al., 2016[72]
MF537248	H33	Japan	Valentin et al., 2017[41]
MF537247	H56	Japan	Valentin et al., 2017[41]
MF537246	H2	China	Valentin et al., 2017[41]
MF537245	H22	China	Valentin et al., 2017[41]
MF537244	H33	China	Valentin et al., 2017[41]
MF537243	H53	Japan	Valentin et al., 2017[41]
MF537242	H69	Japan	Valentin et al., 2017[41]
MF537241	H70	Japan	Valentin et al., 2017[41]
MF537240	H71	Japan	Valentin et al., 2017[41]
MF537239	H72	Japan	Valentin et al., 2017[41]
MF537238	H1017	USA	Valentin et al., 2017[41]
MF537237	H26	China	Valentin et al., 2017[41]
MF537236	H62	Japan, China	Valentin et al., 2017[41]
MF537235	H73	Japan	Valentin et al., 2017[41]
MF537234	H74	Japan	Valentin et al., 2017[41]
MF537233	H75	Japan	Valentin et al., 2017[41]
KY570297	H1017	Canada	Valentin et al., 2017[41]
MF537232	H57	Japan	Valentin et al., 2017[41]
MF537231	H60	Japan	Valentin et al., 2017[41]
MF537230	H61	Japan	Valentin et al., 2017[41]
MF537229	H76	South Korea	Valentin et al., 2017[41]
MF537228	H77	South Korea	Valentin et al., 2017[41]
MF537227	H78	South Korea	Valentin et al., 2017[41]
MF537225	H80	China	Valentin et al., 2017[41]
MF537226	H81	South Korea	Valentin et al., 2017[41]
MF537224	H22	South Korea	Valentin et al., 2017[41]
MF537223	H82	South Korea	Valentin et al., 2017[41]
MF537221	H83	China	Valentin et al., 2017[41]
MF537222	H33	Japan	Valentin et al., 2017[41]
MF537220	H84	South Korea	Valentin et al., 2017[41]
MF537219	H85	Japan	Valentin et al., 2017[41]
KU601523	H43401	Japan	Dhami et al., 2016[72]
KX017333	H24	South Korea	Lee et al., 2018[42]
KU601508	H1	USA	Dhami et al., 2016[72]
KU601509	H1	USA	Dhami et al., 2016[72]
KU601512	H1	China	Dhami et al., 2016[72]

KU601513	H1	USA	Dhami et al., 2016[72]
KU601514	H1	USA	Dhami et al., 2016[72]
MF120271	H86	Switzerland	Morrison et al., 2017[43]
MF120272	H87	Greece	Morrison et al., 2017[43]
MF120273	H33	Greece	Morrison et al., 2017[43]
MF120274	H88	Greece	Morrison et al., 2017[43]
MF120275	H89	Italy	Morrison et al., 2017[43]
KX017397	H1	USA	Lee et al., 2018[42]
KY710424	H53	Italy	Cesari et al., 2018[39]
KY710425	H53	Italy	Cesari et al., 2018[39]
KY710426	H53	Italy	Cesari et al., 2018[39]
KY710427	H53	Italy	Cesari et al., 2018[39]
KY710434	H53	Italy	Cesari et al., 2018[39]
KY710336	H40	Italy	Cesari et al., 2018[39]
KY710346	H40	Italy	Cesari et al., 2018[39]
KY710347	H40	Italy	Cesari et al., 2018[39]
KY710349	H40	Italy	Cesari et al., 2018[39]
KY710359	H40	Italy	Cesari et al., 2018[39]
KY710361	H40	Italy	Cesari et al., 2018[39]
KY710366	H40	Italy	Cesari et al., 2018[39]
KY710372	H40	Italy	Cesari et al., 2018[39]
KY710374	H40	Italy	Cesari et al., 2018[39]
KY710375	H40	Italy	Cesari et al., 2018[39]
KY710376	H40	Italy	Cesari et al., 2018[39]
KY710390	H40	Italy	Cesari et al., 2018[39]
KY710394	H40	Italy	Cesari et al., 2018[39]
KY710325	H54	Italy	Cesari et al., 2018[39]
KY710326	H54	Italy	Cesari et al., 2018[39]
KY710327	H54	Italy	Cesari et al., 2018[39]
KY710355	H54	Italy	Cesari et al., 2018[39]
KY710365	H54	Italy	Cesari et al., 2018[39]
KY710368	H54	Italy	Cesari et al., 2018[39]
KY710369	H54	Italy	Cesari et al., 2018[39]
KY710370	H54	Italy	Cesari et al., 2018[39]
KY710383	H54	Italy	Cesari et al., 2018[39]
KY710385	H54	Italy	Cesari et al., 2018[39]
KY710388	H54	Italy	Cesari et al., 2018[39]
KY710432	H56	Italy	Cesari et al., 2018[39]
KY710450	H56	Italy	Cesari et al., 2018[39]
KY710311	H8	Italy	Cesari et al., 2018[39]
KY710315	H8	Italy	Cesari et al., 2018[39]
KY710316	H8	Italy	Cesari et al., 2018[39]
KY710317	H8	Italy	Cesari et al., 2018[39]
KY710318	H8	Italy	Cesari et al., 2018[39]
KY710319	H8	Italy	Cesari et al., 2018[39]
KY710322	H8	Italy	Cesari et al., 2018[39]
KY710338	H8	Italy	Cesari et al., 2018[39]

KY710340	H8	Italy	Cesari et al., 2018[39]
KY710396	H8	Italy	Cesari et al., 2018[39]
KY710274	H32	Greece	Cesari et al., 2018[39]
KY710279	H32	Greece	Cesari et al., 2018[39]
KY710277	H1	Greece	Cesari et al., 2018[39]
KY710278	H1	Greece	Cesari et al., 2018[39]
KY710281	H1	Italy	Cesari et al., 2018[39]
KY710282	H1	Italy	Cesari et al., 2018[39]
KY710283	H1	Italy	Cesari et al., 2018[39]
KY710284	H1	Italy	Cesari et al., 2018[39]
KY710285	H1	Italy	Cesari et al., 2018[39]
KY710287	H1	Italy	Cesari et al., 2018[39]
KY710288	H1	Italy	Cesari et al., 2018[39]
KY710289	H1	Italy	Cesari et al., 2018[39]
KY710290	H1	Italy	Cesari et al., 2018[39]
KY710291	H1	Italy	Cesari et al., 2018[39]
KY710292	H1	Italy	Cesari et al., 2018[39]
KY710293	H1	Italy	Cesari et al., 2018[39]
KY710295	H1	Italy	Cesari et al., 2018[39]
KY710296	H1	Italy	Cesari et al., 2018[39]
KY710298	H1	Italy	Cesari et al., 2018[39]
KY710299	H1	Italy	Cesari et al., 2018[39]
KY710301	H1	Italy	Cesari et al., 2018[39]
KY710303	H1	Italy	Cesari et al., 2018[39]
KY710304	H1	Italy	Cesari et al., 2018[39]
KY710305	H1	Italy	Cesari et al., 2018[39]
KY710306	H1	Italy	Cesari et al., 2018[39]
KY710309	H1	Italy	Cesari et al., 2018[39]
KY710313	H1	Italy	Cesari et al., 2018[39]
KY710314	H1	Italy	Cesari et al., 2018[39]
KY710324	H1	Italy	Cesari et al., 2018[39]
KY710328	H1	Italy	Cesari et al., 2018[39]
KY710329	H1	Italy	Cesari et al., 2018[39]
KY710330	H1	Italy	Cesari et al., 2018[39]
KY710331	H1	Italy	Cesari et al., 2018[39]
KY710332	H1	Italy	Cesari et al., 2018[39]
KY710333	H1	Italy	Cesari et al., 2018[39]
KY710335	H1	Italy	Cesari et al., 2018[39]
KY710337	H1	Italy	Cesari et al., 2018[39]
KY710343	H1	Italy	Cesari et al., 2018[39]
KY710344	H1	Italy	Cesari et al., 2018[39]
KY710345	H1	Italy	Cesari et al., 2018[39]
KY710348	H1	Italy	Cesari et al., 2018[39]
KY710362	H1	Italy	Cesari et al., 2018[39]
KY710384	H1	Italy	Cesari et al., 2018[39]
KY710386	H1	Italy	Cesari et al., 2018[39]
KY710389	H1	Italy	Cesari et al., 2018[39]

KY710391	H1	Italy	Cesari et al., 2018[39]
KY710395	H1	Italy	Cesari et al., 2018[39]
KY710399	H1	Italy	Cesari et al., 2018[39]
KY710407	H1	Italy	Cesari et al., 2018[39]
KY710408	H1	Italy	Cesari et al., 2018[39]
KY710409	H1	Italy	Cesari et al., 2018[39]
KY710410	H1	Italy	Cesari et al., 2018[39]
KY710411	H1	Italy	Cesari et al., 2018[39]
KY710412	H1	Italy	Cesari et al., 2018[39]
KY710413	H1	Italy	Cesari et al., 2018[39]
KY710414	H1	Italy	Cesari et al., 2018[39]
KY710415	H1	Italy	Cesari et al., 2018[39]
KY710416	H1	Italy	Cesari et al., 2018[39]
KY710417	H1	Italy	Cesari et al., 2018[39]
KY710418	H1	Italy	Cesari et al., 2018[39]
KY710419	H1	Italy	Cesari et al., 2018[39]
KY710420	H1	Italy	Cesari et al., 2018[39]
KY710421	H1	Italy	Cesari et al., 2018[39]
KY710422	H1	Italy	Cesari et al., 2018[39]
KY710423	H1	Italy	Cesari et al., 2018[39]
KY710429	H1	Italy	Cesari et al., 2018[39]
KY710430	H1	Italy	Cesari et al., 2018[39]
KY710437	H1	Italy	Cesari et al., 2018[39]
KY710438	H1	Italy	Cesari et al., 2018[39]
KY710439	H1	Italy	Cesari et al., 2018[39]
KY710440	H1	Italy	Cesari et al., 2018[39]
KY710442	H1	Italy	Cesari et al., 2018[39]
KY710444	H1	Italy	Cesari et al., 2018[39]
KY710445	H1	Italy	Cesari et al., 2018[39]
KY710446	H1	Italy	Cesari et al., 2018[39]
KY710447	H1	Italy	Cesari et al., 2018[39]
KY710448	H1	Italy	Cesari et al., 2018[39]
KY710449	H1	Italy	Cesari et al., 2018[39]
KY710454	H1	Romania	Cesari et al., 2018[39]
KY710455	H1	Romania	Cesari et al., 2018[39]
KY710456	H1	Romania	Cesari et al., 2018[39]
KY710457	H1	Romania	Cesari et al., 2018[39]
KY710458	H1	Romania	Cesari et al., 2018[39]
KY710459	H1	Romania	Cesari et al., 2018[39]
KY710460	H1	Romania	Cesari et al., 2018[39]
KY710286	H3	Italy	Cesari et al., 2018[39]
KY710297	H3	Italy	Cesari et al., 2018[39]
KY710300	H3	Italy	Cesari et al., 2018[39]
KY710302	H3	Italy	Cesari et al., 2018[39]
KY710307	H3	Italy	Cesari et al., 2018[39]
KY710308	H3	Italy	Cesari et al., 2018[39]
KY710310	H3	Italy	Cesari et al., 2018[39]

KY710312	H3	Italy	Cesari et al., 2018[39]
KY710320	H3	Italy	Cesari et al., 2018[39]
KY710321	H3	Italy	Cesari et al., 2018[39]
KY710334	H3	Italy	Cesari et al., 2018[39]
KY710339	H3	Italy	Cesari et al., 2018[39]
KY710341	H3	Italy	Cesari et al., 2018[39]
KY710342	H3	Italy	Cesari et al., 2018[39]
KY710387	H3	Italy	Cesari et al., 2018[39]
KY710397	H3	Italy	Cesari et al., 2018[39]
KY710398	H3	Italy	Cesari et al., 2018[39]
KY710435	H3	Italy	Cesari et al., 2018[39]
KY710443	H3	Italy	Cesari et al., 2018[39]
KY710452	H3	Italy	Cesari et al., 2018[39]
KY710453	H3	Italy	Cesari et al., 2018[39]
KY710272	H33	Greece	Cesari et al., 2018[39]
KY710273	H33	Greece	Cesari et al., 2018[39]
KY710271	H33	Greece	Cesari et al., 2018[39]
KY710275	H33	Greece	Cesari et al., 2018[39]
KY710276	H33	Greece	Cesari et al., 2018[39]
KY710280	H33	Greece	Cesari et al., 2018[39]
KY710323	H52	Italy	Cesari et al., 2018[39]
KY710350	H52	Italy	Cesari et al., 2018[39]
KY710351	H52	Italy	Cesari et al., 2018[39]
KY710352	H52	Italy	Cesari et al., 2018[39]
KY710353	H52	Italy	Cesari et al., 2018[39]
KY710354	H52	Italy	Cesari et al., 2018[39]
KY710356	H52	Italy	Cesari et al., 2018[39]
KY710357	H52	Italy	Cesari et al., 2018[39]
KY710358	H52	Italy	Cesari et al., 2018[39]
KY710360	H52	Italy	Cesari et al., 2018[39]
KY710363	H52	Italy	Cesari et al., 2018[39]
KY710364	H52	Italy	Cesari et al., 2018[39]
KY710367	H52	Italy	Cesari et al., 2018[39]
KY710371	H52	Italy	Cesari et al., 2018[39]
KY710373	H52	Italy	Cesari et al., 2018[39]
KY710377	H52	Italy	Cesari et al., 2018[39]
KY710378	H52	Italy	Cesari et al., 2018[39]
KY710379	H52	Italy	Cesari et al., 2018[39]
KY710380	H52	Italy	Cesari et al., 2018[39]
KY710381	H52	Italy	Cesari et al., 2018[39]
KY710382	H52	Italy	Cesari et al., 2018[39]
KY710392	H52	Italy	Cesari et al., 2018[39]
KY710393	H52	Italy	Cesari et al., 2018[39]
KY710400	H52	Italy	Cesari et al., 2018[39]
KY710401	H52	Italy	Cesari et al., 2018[39]
KY710402	H52	Italy	Cesari et al., 2018[39]
KY710403	H52	Italy	Cesari et al., 2018[39]

KY710404	H52	Italy	Cesari et al., 2018[39]
KY710405	H52	Italy	Cesari et al., 2018[39]
KY710406	H52	Italy	Cesari et al., 2018[39]
KY710441	H52	Italy	Cesari et al., 2018[39]
KY710294	H55	Italy	Cesari et al., 2018[39]
KY710433	H57	Italy	Cesari et al., 2018[39]
KY710431	H58	Italy	Cesari et al., 2018[39]
KY710451	H58	Italy	Cesari et al., 2018[39]
KX100883	H22	native area	Zhu et al., 2016[40]
KX100884	H22	native area	Zhu et al., 2016[40]
KX100885	H24	native area	Zhu et al., 2016[40]
KX100886	H22	native area	Zhu et al., 2016[40]
KX100887	H22	native area	Zhu et al., 2016[40]
KX100888	H1	native area	Zhu et al., 2016[40]
KX100889	H128	native area	Zhu et al., 2016[40]
KX100890	H59	native area	Zhu et al., 2016[40]
KX100891	H24	native area	Zhu et al., 2016[40]
KX100892	H3	native area	Zhu et al., 2016[40]
KX100893	H90	native area	Zhu et al., 2016[40]
KX100894	H7	native area	Zhu et al., 2016[40]
KX100895	H91	native area	Zhu et al., 2016[40]
KX100896	H1	native area	Zhu et al., 2016[40]
KX100897	H17	native area	Zhu et al., 2016[40]
KX100898	H3	native area	Zhu et al., 2016[40]
KX100899	H98	native area	Zhu et al., 2016[40]
KX100900	H92	native area	Zhu et al., 2016[40]
KX100901	H93	native area	Zhu et al., 2016[40]
KX100902	H18	native area	Zhu et al., 2016[40]
KX100903	H14	native area	Zhu et al., 2016[40]
KX100904	H119	native area	Zhu et al., 2016[40]
KX100905	H135	native area	Zhu et al., 2016[40]
KX100906	H3	native area	Zhu et al., 2016[40]
KX100907	H95	native area	Zhu et al., 2016[40]
KX100908	H64	native area	Zhu et al., 2016[40]
KX100909	H96	native area	Zhu et al., 2016[40]
KX100910	H118	native area	Zhu et al., 2016[40]
KX100911	H98	native area	Zhu et al., 2016[40]
KX100912	H14	native area	Zhu et al., 2016[40]
KX100913	H26	native area	Zhu et al., 2016[40]
KX100914	H3	native area	Zhu et al., 2016[40]
KX100915	H22	native area	Zhu et al., 2016[40]
KX100916	H3	native area	Zhu et al., 2016[40]
KX100917	H99	native area	Zhu et al., 2016[40]
KX100918	H100	native area	Zhu et al., 2016[40]
KX100919	H101	native area	Zhu et al., 2016[40]
KX100920	H102	native area	Zhu et al., 2016[40]
KX100921	H103	native area	Zhu et al., 2016[40]

KX100922	H102	native area	Zhu et al., 2016[40]
KX100923	H121	native area	Zhu et al., 2016[40]
KX100924	H126	native area	Zhu et al., 2016[40]
KX100925	H103	native area	Zhu et al., 2016[40]
KX100926	H120	native area	Zhu et al., 2016[40]
KX100927	H1	native area	Zhu et al., 2016[40]
KX100928	H3	native area	Zhu et al., 2016[40]
KX100929	H22	native area	Zhu et al., 2016[40]
KX100930	H136	native area	Zhu et al., 2016[40]
KX100931	H22	native area	Zhu et al., 2016[40]
KX100932	H108	native area	Zhu et al., 2016[40]
KX100933	H109	native area	Zhu et al., 2016[40]
KX100934	H22	native area	Zhu et al., 2016[40]
KX100935	H124	native area	Zhu et al., 2016[40]
KX100936	H1	native area	Zhu et al., 2016[40]
KX100937	H110	native area	Zhu et al., 2016[40]
KX100938	H22	native area	Zhu et al., 2016[40]
KX100939	H115	native area	Zhu et al., 2016[40]
KX100940	H3	native area	Zhu et al., 2016[40]
KX100941	H22	native area	Zhu et al., 2016[40]
KX100942	H83	native area	Zhu et al., 2016[40]
KX100943	H22	native area	Zhu et al., 2016[40]
KX100944	H23	native area	Zhu et al., 2016[40]
KX100945	H33	native area	Zhu et al., 2016[40]
KX100946	H69	native area	Zhu et al., 2016[40]
KX100947	H23	native area	Zhu et al., 2016[40]
KX100948	H123	native area	Zhu et al., 2016[40]
KX100949	H1	native area	Zhu et al., 2016[40]
KX100950	H124	native area	Zhu et al., 2016[40]
KX100951	H112	native area	Zhu et al., 2016[40]
KX100952	H22	native area	Zhu et al., 2016[40]
KX100953	H137	native area	Zhu et al., 2016[40]
KX100954	H1	native area	Zhu et al., 2016[40]
KX100955	H22	native area	Zhu et al., 2016[40]
KX100956	H137	native area	Zhu et al., 2016[40]
KX100957	H22	native area	Zhu et al., 2016[40]
KX100958	H33	native area	Zhu et al., 2016[40]
KX100959	H113	native area	Zhu et al., 2016[40]
KX100960	H52	native area	Zhu et al., 2016[40]
KX100961	H114	native area	Zhu et al., 2016[40]
KX100962	H54	native area	Zhu et al., 2016[40]
KX100963	H40	native area	Zhu et al., 2016[40]
KX100964	H114	native area	Zhu et al., 2016[40]
KX100965	H1	native area	Zhu et al., 2016[40]
KX100966	H22	native area	Zhu et al., 2016[40]
KX100967	H117	native area	Zhu et al., 2016[40]
KX100968	H33	native area	Zhu et al., 2016[40]

KX100969	H22	native area	Zhu et al., 2016[40]
KX100970	H22	native area	Zhu et al., 2016[40]
KX100971	H22	native area	Zhu et al., 2016[40]
KX100972	H33	native area	Zhu et al., 2016[40]
KX100973	H22	native area	Zhu et al., 2016[40]
KX100974	H2	native area	Zhu et al., 2016[40]
KX100975	H2	native area	Zhu et al., 2016[40]
KX100976	H94	native area	Zhu et al., 2016[40]
KX100977	H94	native area	Zhu et al., 2016[40]
KX100978	H1	native area	Zhu et al., 2016[40]
KX100979	H97	native area	Zhu et al., 2016[40]
KX100986	H42	native area	Zhu et al., 2016[40]
KX100987	H105	native area	Zhu et al., 2016[40]
KX100988	H106	native area	Zhu et al., 2016[40]
KX100989	H129	native area	Zhu et al., 2016[40]
KX100990	H22	native area	Zhu et al., 2016[40]
KX100991	H22	native area	Zhu et al., 2016[40]
KX100992	H22	native area	Zhu et al., 2016[40]
KX100993	H111	native area	Zhu et al., 2016[40]
KX100994	H1	native area	Zhu et al., 2016[40]
KX100995	H134	native area	Zhu et al., 2016[40]
KX100996	H24	native area	Zhu et al., 2016[40]
KX100997	H115	native area	Zhu et al., 2016[40]
KX100998	H22	native area	Zhu et al., 2016[40]
KX100999	H22	native area	Zhu et al., 2016[40]
KX101000	H22	native area	Zhu et al., 2016[40]
KX101001	H1	native area	Zhu et al., 2016[40]
KX101002	H120	native area	Zhu et al., 2016[40]
KX101003	H14	native area	Zhu et al., 2016[40]
KX101004	H120	native area	Zhu et al., 2016[40]
KX101005	H1	native area	Zhu et al., 2016[40]
KX101006	H124	native area	Zhu et al., 2016[40]
KX101007	H125	native area	Zhu et al., 2016[40]
KX101008	H14	native area	Zhu et al., 2016[40]
KX101009	H127	native area	Zhu et al., 2016[40]
KX101010	H2	native area	Zhu et al., 2016[40]
KX101011	H129	native area	Zhu et al., 2016[40]
KX101012	H134	native area	Zhu et al., 2016[40]
KX101013	H77	native area	Zhu et al., 2016[40]
KX101014	H132	native area	Zhu et al., 2016[40]
KX101015	H22	native area	Zhu et al., 2016[40]
MK779996	H162	Italy	Kapantaidaki et al., 2019[37]
MK779997	H1	Italy	Kapantaidaki et al., 2019[37]
KY379175	P1	USA, China	Otero-Bravo & Sabree, 2018[3]
KY379176	P2	USA, China, Switzerlnad	Otero-Bravo & Sabree, 2018[3]
AP012554	P2	USA	Kobayashi et al., unpublished

Figure S1. Parsimony network built with TCS 1.21 using our data (this study) and data from the NCBI dataset, which shows the relationships occurring among *Halyomorpha halys* different haplotypes. The colours represent different geographic distribution. Each haplotype is represented by a circle, and the area of the circle is proportional to its frequency. Small black dots symbolize missing intermediate or unsampled haplotypes. Asterisk * indicates new haplotypes found in this study.

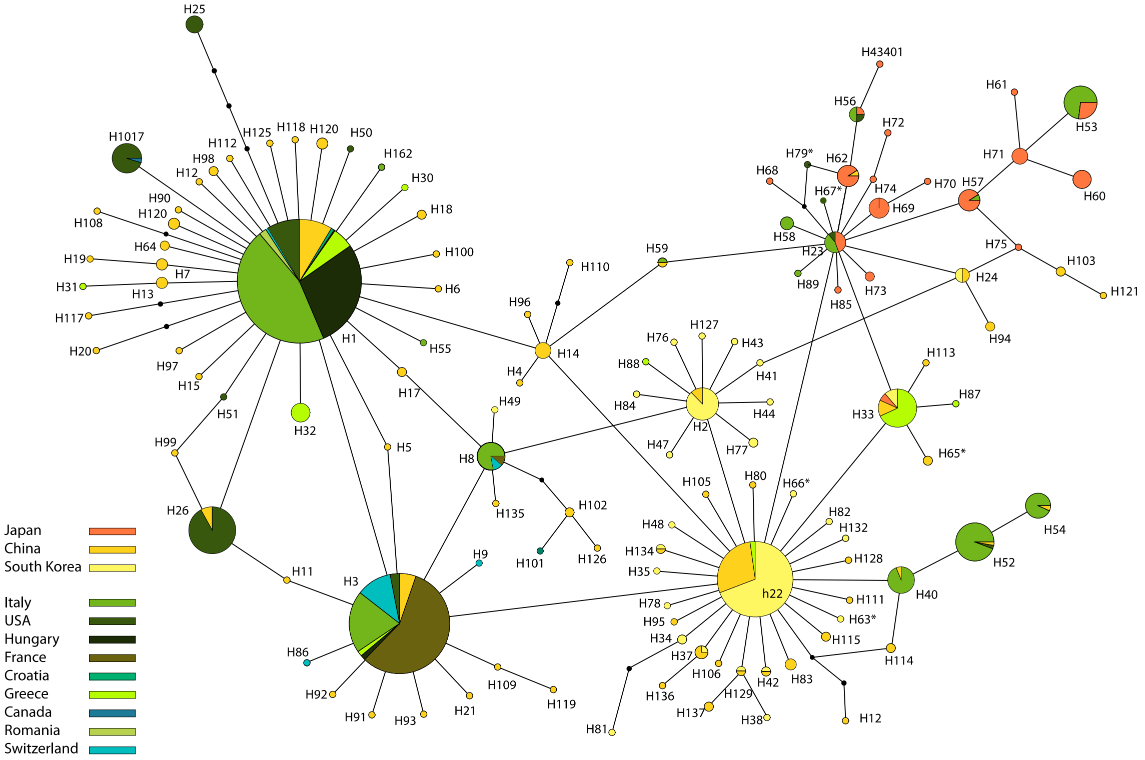

Figure S2. Rarefaction analysis of *Halyomorpha halys* sequences considering the COI marker. Rarefaction curves were calculated considering the number of OTUs obtained at the 0% level of sequence divergence.

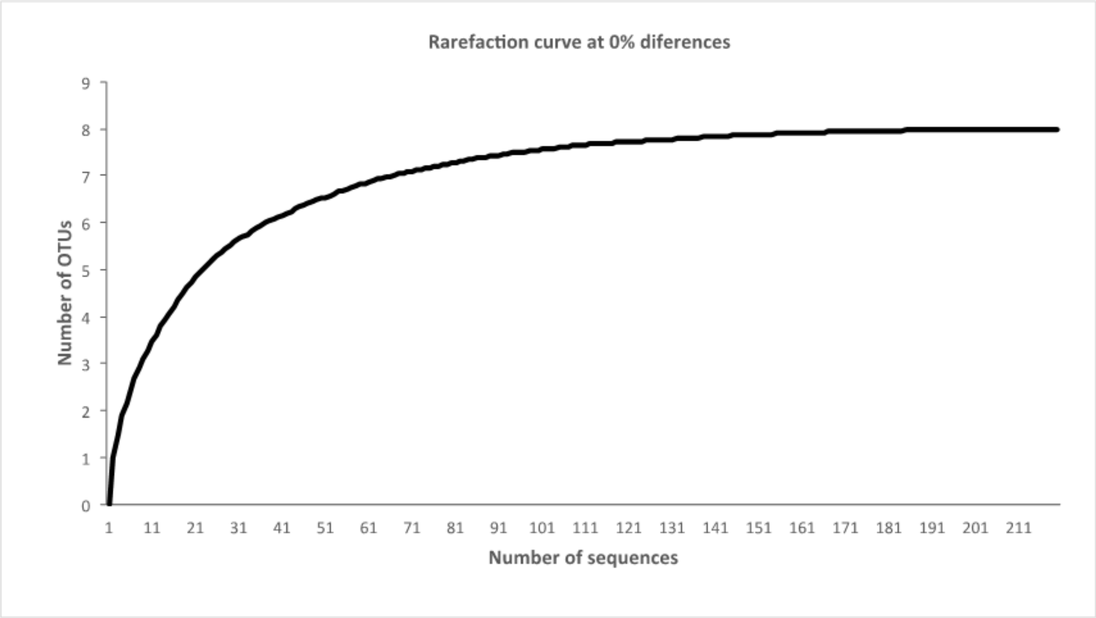

Table S2. Neutrality tests values (Tajima's D and Fu's FS) in *H. halys* populations within countries. Asterisk * indicates $p < 0.05$.

	**Tajima's D**	**Fu's Fs**
**Italy**	0.273	-0.563
**France**	-1.470*	-2.822*
**Greece**	-0.188	-0.772
**Hungary**	-0.702	-0.790
**USA**	-1.233	-2.511
**China**	-1.669*	-22.77*
**Japan**	-0.933	-8.707*
**South Korea**	-2.149*	-26.34*
**Switzerland**	-1.592*	-3.338*
**Romania**	0.000	0.000
**Croatia**	0.000	0.000
**Canada**	0.000	0.000

Figure S3. Mismatch distribution under the population expansion model of native and newly invaded *H. halys* populations within countries based on pairwise differences. The sums of squared deviations (SSD), raggedness index (r) and their corresponding P-values are given.

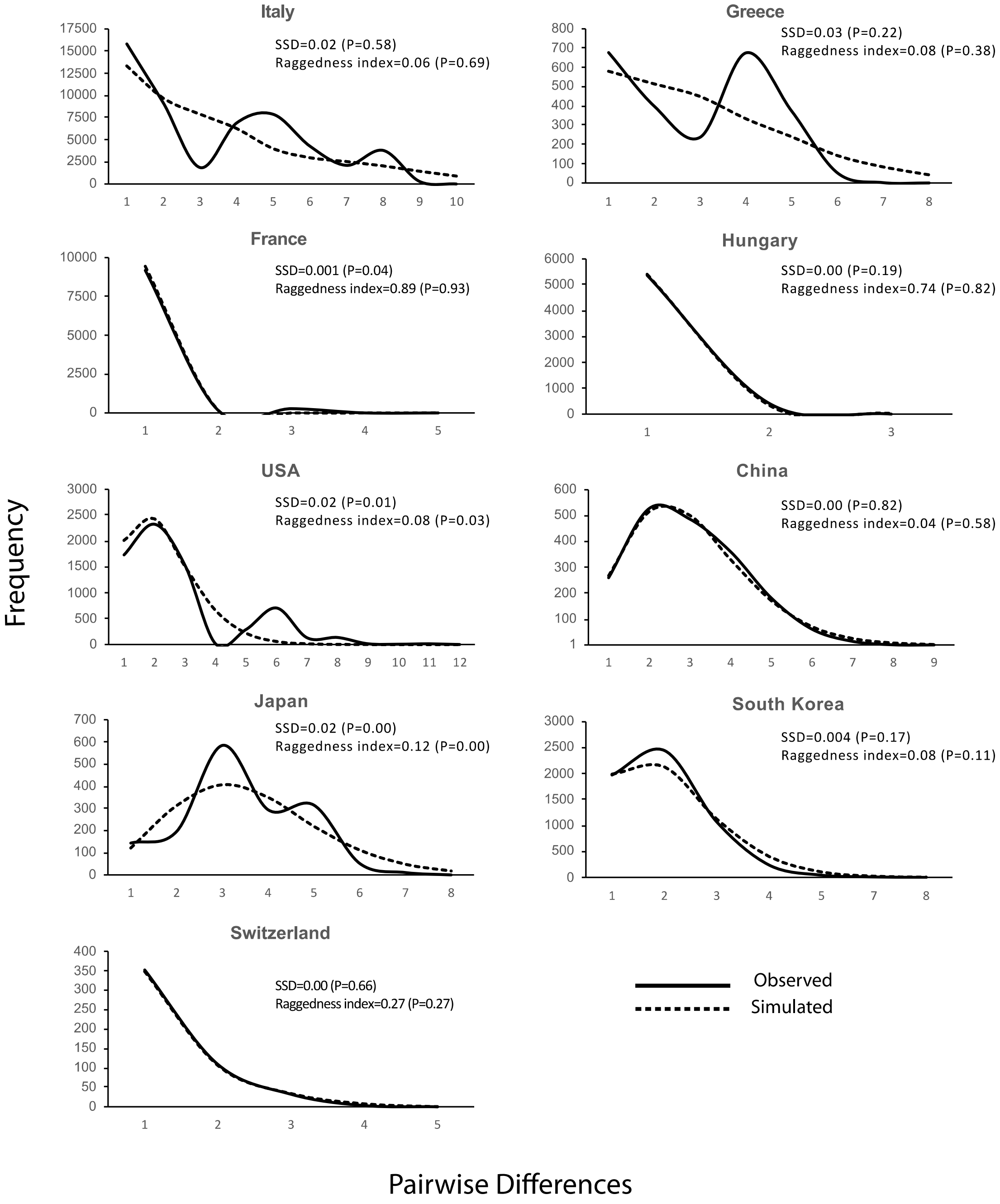

Supplement: Supplementary file 1 — Supplementary Information [file 41598_2020_75519_MOESM1_ESM.pdf]
